# The nonlinear effect of financial and fiscal policies on poverty alleviation in China—An empirical analysis of Chinese 382 impoverished counties with PSTR models

**DOI:** 10.1371/journal.pone.0224375

**Published:** 2019-11-05

**Authors:** Xicong Kuang, Huihuang Liu, Guoqiang Guo, Haixing Cheng

**Affiliations:** 1 School of Economics and Trade, Hunan University, Changsha, China; 2 School of Economics, Huazhong University of Science and Technology, Wuhan, China; 3 School of Economics, Huazhong University of Science and Technology, Wuhan, China; The Bucharest University of Economic Studies, ROMANIA

## Abstract

Using panel smooth transition regression (PSTR) models, this paper studies the effects of financial and fiscal policies on poverty reduction across 382 poverty-stricken counties in China. The findings are that both fiscal and financial policies have a positive influence on poverty reduction, and their relationships are nonlinear. For either a high or low degree of poverty, fiscal policies are effective for poverty reduction, while financial policies have a greater impact on poverty reduction when there is a medium degree of poverty. Therefore, in deciding which policies should be prioritized for reducing poverty, the level of poverty should be taken into account. To be more specific, when a portfolio of poverty-reduction policies is implemented, fiscal policies should be prioritized at the beginning, when the incidence of poverty is high. Then, financial support should come to the forefront as the poverty level drops, and fiscal support should be stepped up when the poverty level continues to drop.

## Introduction

Promoting the development of poverty-stricken areas to eliminate poverty and achieve common prosperity is the shared aspiration of all mankind. As the most populous developing country in the world, China suffers from a weak economic foundation and uneven development. Poverty-stricken areas in China particularly bears the brunt with a large poor population, making it more difficult to reduce poverty there. Poverty reduction has always been high on the agenda of Chinese policymakers, in 1986, the Chinese government established the Leading Group of Economic Development in poverty-stricken areas (later renamed the State Council Leading Group Office of Poverty Alleviation and Development, referred to as the CPAD), which have been using multiple channels to promote development and raise income levels in impoverished regions.According to the CPAD, the Chinese government has identified the 832 counties as poverty-stricken counties at the end of 2014, thanks to a package of fiscal, taxation, industrial and financial policies, the incidence of rural poverty in China fell from 97.5 percent in 1978 to 1.7 percent in 2018. At present, the poor mainly live in economically backward regions; our country's 832 impoverished counties are largely distributed in economically underdeveloped areas in the Midwest. In particular, 74% of the poverty counties are in the west, and number of poor population in the western region accounted for 84.3% of the total number of poor population across the country. Based on figures released by the National Bureau of Statistics and the “China rural poverty monitoring Report”, according to the author's calculation the correlation coefficient between the poverty incidence and rural per capita income is approximately -0.368.Therefore, One way to reduce poverty is to promote economic development in poor areas. Not only does practice show economic development is the answer to reducing poverty but also plenty of theories prove this is so (Li Xiaoyun et al., 2010; Xia Qingjie et al., 2010; Lin Boqiang, 2003; Zhang Cui, 2011; Hu Bing et al., 2007; Wan Guanghua and Zhang Yin, 2006; Klasen and Waibel,2010; David Dollar and Aart Kraay, 2002; Ravallion,1995; Chambers et al.,2008)[1–10].To achieve economic development in impoverished areas, according to Walt Rostow's Stages of Growth in Economies, three preconditions must be in place: (a.) Increase the rate of productive investment, so that accumulation accounts for more than 10% of national income; (b.) More than one important industry is established and developed; and (c.) institutional reform gives rise to a new political or social structure that allows for the expansion of modern sectors. In reality, the third condition of economic take-off is very difficult, the most important is to achieve economic take-off through the first two conditions.Both improving productivity and developing important industries require capital input, which is essential for economic development in poor areas(Li Xueping, 2015; Jia Yu, 2012; Li Xunlai, Li Guoping, Li Fuzhu, 2005; Zhou Xiao, Zhunong, 2003)[11–14]. This is also true at the micro level: for an individual to recover from poverty, production factors such as land, labor and capital are needed. Economic theories also show that capital is indispensable for an economy to grow fast and reach the state of equilibrium; this is especially true with impoverished areas where the absence of capital is the main obstacle to economic growth and income increase(Liu Wei, Fan Xin, 2019; Xie Danyang, Zhou Zexi, 2019; Wang Chunchao, 2011)[15–17]. All this considered, ramping up capital input is key to economic growth, which leads to poverty reduction.

At present, in poor areas or poor individuals with poor initial conditions, it is very slow and difficult to gradually realize capital accumulation through primitive means, that is, relying on self-cycle. (Xu Changsheng 1992, 1995; Research Team of Agricultural Economics, Research Center of Ministry of Agriculture, 1997; Todaro, 1991; Solow, 1957)[18–22]. Therefore, it is necessary to develop policies including the introduction of capital to speed up capital accumulation, shorten the primitive stage of development and shake off poverty quickly. Fiscal and financial policies are currently the most common methods used to introduce much-needed capital from outside. Many scholars have done empirical researchs on this subject(Cepparulo et al.,2017)[23].It has been proven that both types of policies are effective in boosting economic growth (Yang Youcai, 2014; Wu Zhi, 2010; Li Yankai and Han Yanchun, 2013; Chen Yulu et al., 2016; Wang Dingxiang et al., 2009; Ding Zhiguo et al., 2014; King and Levine, 1993)[24–30]. According to our research, both fiscal and financial policies can positively affect economic growth and increase incomes. However, the relationship is nonlinear and subject to a threshold effect, so a well-designed portfolio combining fiscal and financial policies should be put in place to effectively reduce poverty.

The rest of this paper is structured as follows: Section 2 briefly reviews the literature on the poverty-reduction effects of fiscal and financial policies. Section 3 explains the PSTR models. Section 4 describes the model estimation and interprets the results, and section 5 provides the concluding remarks and policymaking recommendations.

## Theoretical analysis and hypotheses

Financial policies for poverty alleviation include investing in credit and providing financial services to individuals and businesses, such as depositing funds, lending funds, currency exchange, online payments, cash services, and mobile banking. Fiscal policy include transferring payments from the central government, Central Government’s Special Fund and so on. Fiscal and financial policies, as two important ways of introducing capital and fuel economy in poor areas, differ in terms of their effects on poverty reduction.

Financial channels provide more opportunities to access funds, such as idle money collected by banks, grants from central banks or funds generated from interbank business. Some banks can resort to other methods of fund-raising, such as issuing bonds and going public. However, the main money source of fiscal policy is the revenue of local governments and transfer payments from the central government. Although some local governments also issue bonds to raise money from the wider community, the bond-issuing scale is restricted by future revenues and the necessity to balance budgets, hence limiting the amount of money that can be raised. Additionally, the Central Government’s Special Fund for Poverty Alleviation will never be sufficient, and the annual poverty-reduction fund provided by the Chinese central government is only CNY 100 billion.

Regarding their working mechanism, banking institutions are for-profit market entities. The delivery of financial support for economic development and poverty reduction is mainly dictated by the force of the market. Nevertheless, financial resources can be reused and recycled. Enterprises and individuals can obtain continuous financial support, which is effective in the long run. The main body of fiscal expenditure is the government, not for the purpose of profit, the way of action is more inclined to plan. Limited by amount, fiscal policy support cannot be used without limit and recycle.Generally speaking, for poor areas, the use cost of fiscal expenditure is lower than that of financial channels, but the scale and sustainability of fiscal input are worse than that of financial channels.

Currently, the academic community lacks consensus on the actual effect of finance in promoting economic development and poverty reduction. Some believe that finance can alleviate poverty. Cui Yanjuan and Sun Gang (2012)[31] analyze China’s cross-province plane data between 1978 and 2010 and find that financial policies can elevate the income level of the have-nots by galvanizing the local economy and redistributing incomes. However, financial volatility may offset its poverty-reduction effect. Some hold that financial policies may inhibit poverty reduction. Yang Jun et al. (2008)[32] use a vector regression model to prove that financial development in rural areas facilitates poverty reduction in the short run, although the effect is not obvious. Financial development can inhibit poverty reduction in the long run. However, clearly, there is no Granger causality between the two. Wang Xiaohua (2014)[33] also substantiates that the effect of rural credit on increasing income levels is remarkable in nonimpoverished rural areas but limited in impoverished ones.

Regarding the features of finance’s effect on poverty reduction, some scholars hold that the effect is nonlinear. Cui Yanjuan and Sun Gang (2012)[31] prove that financial development will aggravate poverty before alleviating it due to the high cost of providing financial services. Su Jing et al. (2013)[34] use PSTR to prove that unofficial financial services have a nonlinear effect on poverty incidence, depth and intensity.

Regarding the effect of fiscal policies on poverty reduction, some argue that fiscal expenditures can contribute to poverty reduction partly. For instance, Wang Juan and Zhang Kezhong(2012)[35] analyze cross-province plane data between 1994 and 2004 and conclude that social relief expenditures, capital construction expenditures and public spending on rural development can markedly reduce poverty. However, spending on science, education, culture and health care is not as effective for poverty reduction. Having analyzed the effect of fiscal decentralization between 1995 and 2010 on rural poverty, Chu Deyin and Zhaofei (2013)[36] find that the increase of decentralization of budgetary income and expenditure is beneficial to alleviate rural poverty, but the increase of decentralization of extrabudgetary income worsens rural poverty. Zhang Kezhong et al. (2010)[37] analyze cross-province data after tax reform and find that fiscal decentralization will exacerbate poverty in Beijing, Shanghai and Tianjin but reduce poverty in other regions. Some scholars argue that fiscal expenditures have no obvious effect on poverty alleviation. Li Yongyou and Shen Kunrong (2007)[38] study data from 1980–2005 and conclude that relative poverty in China is mainly caused by price differences in the labor force of different professions at the initial stage of wealth distribution. Therefore, fiscal policies have little impact on alleviating poverty caused by differences in labor force prices. Moreover, expenditures on health care will even worsen poverty.

On the other hand, most of the previous research focuses on the effect of financial and fiscal policies in reducing poverty based on cross-province data. Few studies focus on smaller units, such as impoverished counties. As China’s economy grows, the official identification of the smallest poverty unit moves up to the county level.

Building on previous research, especially on the working mechanism and difference between the effects of financial and fiscal policies, this paper proposes 2 hypotheses:

H1. Both financial and fiscal policies are important for poverty reduction. However, the relationship is nonlinear, meaning the effectiveness of both policies differ from stage to stage.
H2. Financial and fiscal measures are complementary in reducing poverty, meaning the poverty alleviating effect of both policies vary and complement each other at different stages of poverty. A reasonable use of financial and fiscal policies contributes to effective poverty reduction.

## Modeling and data processing

### Specification of the model

The PSTR model (Gonazlez A. et al., 2017)[39] analyzing the effects of financial polices on poverty reduction is as follows:

$$Y_{it}=\mu_{i}^{b}+\beta_{1}^{b}BK_{it}+\beta_{2}^{b}G_{it}+(\beta_{3}^{b}BK_{it}+\beta_{4}^{b}G_{it})h_{2}(q_{it}^{b};\gamma^{b},c^{b})+\varepsilon_{it}^{b}$$ (1)

The PSTR model that analyzes the effects of fiscal policies on poverty reduction is as follows:

$$Y_{it}=\mu_{i}^{f}+\beta_{1}^{f}BK_{it}+\beta_{2}^{f}G_{it}+(\beta_{3}^{f}BK_{it}+\beta_{4}^{f}G_{it})h_{2}(q_{it}^{f};\gamma^{f},c^{f})+\varepsilon_{it}^{f}$$ (2)

*Y*_*it*_, *BK*_*it*_, *FK*_*it*_, and *G*_*it*_ denote the level of economic development in poor regions, the strength of financial support, the strength of fiscal support and the level of fixed asset investment, respectively. $\mu_{i}^{b}$ and $\mu_{i}^{f}$ represent differentiated individual specific effects of poor regions. $\varepsilon_{it}^{b}$ and $\varepsilon_{it}^{f}$ are random disturbance terms. $q_{it}^{b}$ and $q_{it}^{f}$ stand for transformed variables. The degree of poverty is selected as the conversion variable. $h_{z}(q_{it}^{b};\gamma^{b},c^{b})$ and $h_{z}(q_{it}^{f};\gamma^{f},c^{f})$ are continuous, bounded transition functions for $q_{it}^{b}$ and $q_{it}^{f}$ and satisfy $0\leq h_{z}(q_{it}^{b};\gamma^{b},c^{b})\leq 1$ and $0\leq h_{z}(q_{it}^{f};\gamma^{f},c^{f})\leq 1$. The logistic functions of $h_{z}(q_{it}^{b};\gamma^{b},c^{b})$ and $h_{z}(q_{it}^{f};\gamma^{f},c^{f})$ are as follows:

$$h_{z}(q_{it}^{b};\gamma^{b},c^{b})=[1+e^{-\gamma^{b}\sum_{z=1}^{M}\left(q_{it}^{b}-c_{z}^{b}\right)}{]}^{-1}$$ (3)

$$h_{z}(q_{it}^{f};\gamma^{f},c^{f})=[1+e^{-\gamma^{f}\sum_{z=1}^{N}\left(q_{it}^{f}-c_{z}^{f}\right)}{]}^{-1}$$ (4)

*γ*^*f*^>0; $c_{1}^{b}<c_{2}^{b}<\ldots <c_{M}^{b}$; and $c_{1}^{f}<c_{2}^{f}<\ldots <c_{N}^{f}$. $c_{i}^{b}$ represents the locations where the transition of the effects of financial policies occur; $c_{j}^{f}$ denotes the location where the transition of the effects of financial policies occur, which is dubbed “the threshold level” determining where the transition of the effects of financial or fiscal policies should happen. *γ*^*b*^ and *γ*^*f*^ represent the smooth parameters, determining the smoothness of the transitions. The higher the value of *γ* is, the faster or more acute the transition takes place and vice versa. When the value is approaching 0, the nonlinear model will be nearing a linear model. M represents the location parameter in the transition function $h_{z}(q_{it}^{b};\gamma^{b},c^{b})$ for the effects of financial policies, meaning that the transition of finance’s poverty reduction effect will happen at the location M. Similarly, N represents the location parameter in the transition function $h_{z}(q_{it}^{f};\gamma^{f},c^{f})$ for the effects of fiscal policies, meaning that the transition of the poverty-reduction effect of fiscal policies will happen at location N. Normally, the value of M or N is 1 or 2, indicating that there will be 1–2 transition locations; when *M* = *N* = 1, it implies that the transition of poverty-reduction effect in fiscal or financial model will occur at only one location and the transition functions $h_{z}(q_{it}^{b};\gamma^{b},c^{b})$ and $h_{z}(q_{it}^{f};\gamma^{f},c^{f})$ will change to

$$h_{1}(q_{it}^{b};\gamma^{b},c^{b})=[1+e^{-\gamma^{b}(q_{it}^{b}-c^{b})}{]}^{-1}$$ (5)

$$h_{1}(q_{it}^{f};\gamma^{f},c^{f})=[1+e^{-\gamma^{f}(q_{it}^{f}-c^{f})}{]}^{-1}$$ (6)

It is noticeable that $\underset{q_{it}^{b}\to -\infty }{\lim}h_{1}(q_{it}^{b};\gamma^{b},c^{b})=0$, $\underset{q_{it}^{f}\to -\infty }{\lim}h_{1}(q_{it}^{f};\gamma^{f},c^{f})=0$, $\underset{q_{it}^{b}\to +\infty }{\lim}h_{1}(q_{it}^{b};\gamma^{b},c^{b})=1$, and $\underset{q_{it}^{f}\to +\infty }{\lim}h_{1}(q_{it}^{f};\gamma^{f},c^{f})=1$. When $\underset{q_{it}^{b}\to -\infty }{\lim}h_{1}(q_{it}^{b};\gamma^{b},c^{b})=0$, the poverty-reduction effect of financial policies is in the low regime; when $\underset{q_{it}^{f}\to -\infty }{\lim}h_{1}(q_{it}^{f};\gamma^{f},c^{f})=0$, the poverty-reduction effect of fiscal policies is in the low regime. Conversely, when $\underset{q_{it}^{b}\to +\infty }{\lim}h_{1}(q_{it}^{b};\gamma^{b},c^{b})=1$, the poverty-reduction effect of financial policies is in the high regime, and when $\underset{q_{it}^{f}\to +\infty }{\lim}h_{1}(q_{it}^{f};\gamma^{f},c^{f})=1$, the poverty-reduction effect of fiscal policies is in the high regime. When *M* = *N* = 2, the transition of the poverty-reduction effect in the fiscal or financial model will occur at 2 locations, and the transition functions $h_{z}(q_{it}^{b};\gamma^{b},c^{b})$ and $h_{z}(q_{it}^{f};\gamma^{f},c^{f})$ will change to

$$h_{2}(q_{it}^{b};\gamma^{b},c^{b})=[1+e^{-\gamma b(q_{it}^{b}-c_{1}^{b})(q_{it}^{b}-c_{2}^{b})}{]}^{-1}$$ (7)

$$h_{2}(q_{it}^{f};\gamma^{f},c^{f})=[1+e^{-\gamma^{f}(q_{it}^{f}-c_{1}^{f})(q_{it}^{f}-c_{2}^{f})}{]}^{-1}$$ (8)

In this case, $h_{2}(q_{it}^{b};\gamma^{b},c^{b})$ and $h_{2}(q_{it}^{f};\gamma^{f},c^{f})$ are the quadratic functions of $q_{it}^{b}$ and $q_{it}^{f}$. Meanwhile, $h_{2}(q_{it}^{b};\gamma^{b},c^{b})$ is symmetrical at both sides of $q_{it}^{b}=\frac{\left(c_{2}^{b}+c_{2}^{b}\right)}{2}$ and reaches the minimum value at $q_{it}^{b}=\frac{\left(c_{1}^{b}+c_{2}^{b}\right)}{2}$; $h_{2}(q_{it}^{f};\gamma^{f},c^{f})$ is symmetrical at both sides of $q_{it}^{f}=\frac{\left(c_{2}^{f}+c_{2}^{f}\right)}{2}$ and reaches the minimum value at $q_{it}^{f}=\frac{\left(c_{1}^{f}+c_{2}^{f}\right)}{2}$.

In the PSTR model, the marginal effect of financial and fiscal support on poverty reduction is given by

$$u_{it}^{b}=\frac{\partial Y_{it}}{\partial BK_{it}}=\beta_{1}^{b}+\beta_{3}^{b}h_{z}(q_{it}^{b};\gamma^{b},c^{b})$$ (9)

$$u_{it}^{f}=\frac{\partial Y_{it}}{\partial FK_{it}}=\beta_{1}^{f}+\beta_{3}^{f}h_{z}(q_{it}^{f};\gamma^{f},c^{f})$$ (10)

In the nonlinear models, the poverty-reduction effects of fiscal and financial policies are dynamic, not fixed. Given $0\leq h_{z}(q_{it}^{b};\gamma^{b},c^{b})\leq 1$, when the valuation of $\beta_{3}^{b}$ is over 0, the marginal effect of financial policies on poverty reduction increases as the transition functions increase and diminishes as the transition functions diminish. The same rule also applies to the marginal effect of fiscal policies on poverty reduction.

The empirical study in this paper is divided into two steps. At the first step, in order to visualize and intuitively see the effects of financial and fiscal policy on the level of economic development under different mechanisms, we artificially categorize the incidence of poverty from low to high; the categories are 0–5%, 5–10%, 10–15%, 85–90% and 90–100% and are divided into 19 sections. We use data from each segment to establish the linear relationships among per capita GDP, urban per capita income, rural per capita income, per capita loans, per capita fiscal expenditures and per capita fixed asset investment(see 4.2). At the second step, in order to more accurately describe the mechanism of financial and fiscal policy, the nonlinear panel smooth transformation model is adopted to estimate the relationship between variables(see 4.3).

### 3.2 The selection and processing of the dataset

This paper initially collected relevant information on 832 poverty-stricken counties, but data on some poverty-stricken counties were seriously lacking. In the end, there were 382 poverty-stricken counties with complete data. Therefore, we selected 382 poverty-stricken counties out of 832 poverty-stricken counties in the nation as research objects and empirical analysis objects, with a focus on the effects of financial and fiscal policies on economic development and thus poverty reduction from 2010 to 2015. Table 1 shows the distribution of poverty-stricken counties.

**Table 1:** Distribution of 382 poverty-stricken counties.

	Province	Number of poverty-stricken counties	Total population of poverty-stricken counties(ten thousand)	Number of poor population(ten thousand)	Incidence of poverty(%)
**Eastern region**	Hebei	17	635.6	111.5	17.5
**Central region**	Anhui	12	1438.8	141.1	9.8
	Henan	17	1517.5	163.3	10.8
	Heilongjiang	10	426.7	75.8	17.8
	Hubei	20	1005	181.8	18.1
	Hunan	24	1320	192.3	14.6
	Jilin	2	79	11.2	14.1
	Jiangxi	15	920.6	115.2	12.5
**western region**	Gansu	47	1592.6	285.9	18
	Guangxi	25	861.9	213.8	24.8
	Guizhou	29	1376.9	273.8	19.9
	Neimenggu	7	216.4	30.2	14
	Ningxia	5	132.2	32.1	24.3
	Qinghai	17	239.7	52.1	21.7
	Shānxi	9	196.9	47.1	23.9
	Shǎnxi	30	863.7	201.6	23.3
	Sichuan	45	1380	233.9	16.9
	Xizang	5	30	2.4	8.1
	Xinjiang	16	509.5	92.4	18.1
	Yunnan	21	1160.1	245.6	21.2
	Chongqing	9	572	57.8	10.1
**Total**		382	16475.1	2760.8	16.8

#### Degree of poverty

There are multiple dimensions and indicators at hand to assess the degree of poverty, including the local income level, the impoverished population and the incidence of poverty. To make the poverty indicators of 382 impoverished counties comparable, this paper uses the incidence of poverty to evaluate the degree of poverty (Wang Rongdang, 2006; Yu Fangdong, 2004; Shen Xuemin, 1986)[40–42]. We calculates the quarterly index value of poverty incidence from 2010–2015, Table 2 shows the statistical characteristics of the poverty incidence indicators.

**Table 2:** Statistical characteristics of each variable index.

	Unit	N, T	Mean	Median value	Maximum	Minimum	Standard error
**GDP per capita**	yuan/per capita	382, 6	15,097.76	13,131.39	108,985.20	129.31	9308.26
**Urban income per capita**	yuan/per capita	382, 6	16,293.92	16,157.08	30,960	1110.68	4713.56
**Rural income per capita**	yuan/per capita	382, 6	5163.19	4907	12,705.33	414	1759.6
**Incidence of poverty**	%	382, 6	0.269	0.24	0.982	0.002	0.162
**Loans per capita**	yuan/per capita	382, 6	9025.49	6803.19	90,876.87	79.51	8599.7
**Public financial expenditures per capita**	yuan/per capita	382, 6	6611.02	4940.29	59,080.24	36.64	5834.91
**Fixed asset investment per capita**	yuan/per capita	382, 6	16,624.23	12,800.24	219,343.90	657.3427	14,148.13

Note: Data were from National bureau of statistics and the author’s collection and calculation.

#### Economic development level in poor regions

There are many indicators available to evaluate the level of economic development. To assess the economic development of a given region, we chooses per capita GDP, urban per capita income and rural per capita income, which are also important for poverty eradication.

#### Financial support for the poor

There are many financial policies available to support poor regions, such as investing in credit and providing financial services to individuals and businesses, including depositing funds, lending funds, currency exchange, online payments, cash services, and mobile banking. We chooses one of the key financial policies, per capita loans, to assess financial support for poor regions. (Cui Yanjuan, Sun Gang 2012; Su Jing, 2013)[31,34]

#### Fiscal support for the poor

To demonstrate the impact of fiscal expenditures on poverty alleviation and remove the difference in fiscal bases, we uses per capita fiscal expenditures to evaluate fiscal support for poor regions (Qin Jianjun and Wu Laping, 2011; Lin Boqiang, 2003)[43,3].

#### Other explanatory variables

Although this paper focuses on the effects of financial and fiscal policies on promoting economic development and reducing poverty, fixed asset investment has also been included in the model as one of the explanatory variables.

## Model estimation and interpretation of the results

### Stationary test

IPS is used to test the stationary of the numerical variables above, and the results are as follows (Table 3):

**Table 3:** The stationary test results of each variable index.

		IPS test value	P value	1% critical value	5% critical value	10% critical value	Results
**GDP per capita**	Level	3.315	0.999	-4	-3.13	-2.9	Level unstationary, First-order difference stationary
	First-order difference	-10.775	0	18.44	18.733	18.999	
**Urban income per capita**	Level	2.005	0.977	-4	-3.13	-2.9	Level unstationary, First-order difference stationary
	First-order difference	-12.417	0	18.44	18.733	18.999	
**Rural income per capita**	Level	-0.037	0.485	-4	-3.13	-2.9	Level unstationary, First-order difference stationary
	First-order difference	-19.371	0	18.44	18.733	18.999	
**Loans per capita**	Level	3.014	0.998	-4	-3.13	-2.9	Level unstationary, First-order difference stationary
	First-order difference	-11.957	0	18.44	18.733	18.999	
**Public financial expenditures per capita**	Level	1.935	0.973	-4	-3.13	-2.9	Level unstationary, First-order difference stationary
	First-order difference	-14.849	0	18.44	18.733	18.999	
**Fixed asset investment per capita**	Level	-1.403	0.08	-4	-3.13	-2.9	Level unstationary, First-order difference stationary
	First-order difference	-21.099	0	18.44	18.733	18.999	

Table 3 shows that all the variables are non-stationary at the horizontal level but stationary at First order difference. By First order difference for all variables, this paper creates the stationary variables.

### Estimation and results of the piecewise linear regression model

According to Table 4, the estimation of Serial Nos. (1)—(11) shows that when the incidence of poverty is between 15–55%, per capita loans will have a positive impact on per capita GDP; however, the p value is low. The estimation of Serial Nos. (20)–(28) and (35)–(37) indicates that when the incidence of poverty is between 0–45% and 75–90%, per capita fiscal expenditures will have a positive impact on per capita GDP; however, the p value is low.

**Table 4:** Linear relationship between per capita GDP (dependent variable) and the explanatory variables under different poverty incidence.

Incidence of poverty	Serial number	Explanatory variables		Adjusted R squared	Serial number	Explanatory variables		Adjusted R squared
		Loans per capita	Fixed asset investment per capita			Public Financial expenditure per capita	Fixed asset investment per capita	
**0–5%**	(1)	0.014(0.918)	0.25(0.000)	0.193	(20)	0.819(0.023)	0.204(0.001)	0.249
**5–10%**	(2)	0.198(0.005)	0.55(0.000)	0.514	(21)	0.484(0.000)	0.474(0.000)	0.542
**10–15%**	(3)	0.135(0.028)	0.585(0.000)	0.441	(22)	1.017(0.000)	0.361(0.000)	0.593
**15–20%**	(4)	0.14(0.001)	0.409(0.000)	0.462	(23)	0.071(0.408)	0.444(0.000)	0.445
**20–25%**	(5)	0.155(0.003)	0.486(0.000)	0.539	(24)	0.072(0.38)	0.516(0.000)	0.527
**25–30%**	(6)	0.472(0.000)	0.133(0.000)	0.418	(25)	0.405(0.000)	0.171(0.000)	0.304
**30–35%**	(7)	0.281(0.000)	0.125(0.000)	0.258	(26)	0.425(0.000)	0.159(0.000)	0.271
**35–40%**	(8)	0.465(0.000)	0.258(0.000)	0.617	(27)	0.652(0.000)	0.303(0.000)	0.52
**40–45%**	(9)	0.824(0.000)	0.25(0.001)	0.646	(28)	0.918(0.000)	0.426(0.000)	0.463
**45–50%**	(10)	0.315(0.000)	0.275(0.000)	0.722	(29)	-0.045(0.542)	0.435(0.000)	0.637
**50–55%**	(11)	0.523(0.000)	0.318(0.000)	0.721	(30)	-0.224(0.281)	0.535(0.000)	0.422
**55–60%**	(12)	0.236(0.427)	0.7(0.000)	0.558	(31)	0.183(0.158)	0.737(0.000)	0.577
**60–65%**	(13)	-0.128(0.904)	0.349(0.091)	0.674	(32)	0.076(0.822)	0.298(0.086)	0.676
**65–70%**	(14)	0.009(0.905)	0.182(0.01)	0.641	(33)	-0.085(0.64)	0.216(0.01)	0.647
**70–75%**	(15)	0.089(0.763)	0.145(0.285)	0.831	(34)	-0.12(0.308)	0.241(0.003)	0.851
**75–80%**	(16)	1.117(0.000)	-0.116(0.041)	0.827	(35)	0.857(0.012)	-0.063(0.62)	0.258
**80–85%**	(17)	-0.054(0.89)	0.156(0.101)	0.158	(36)	0.275(0.074)	-0.005(0.959)	0.448
**85–90%**	(18)	0.21(0.47)	0.033(0.471)	0.205	(37)	0.16(0.029)	0.013(0.656)	0.491
**90–100%**	(19)	1.343(0.283)	-0.132(0.506)	-0.04	(38)	-0.183(0.166)	0.281(0.166)	0.16

Note: P is the estimated value and is used hereafter.

According to Table 5, the estimation of Serial Nos. (42)—(46) shows that when the incidence of poverty is between 15–40%, per capita loans will positively affect urban per capita income; the p value is low. The estimation of Serial No. (58)–(65) and (72)–(73) indicates that when the incidence of poverty is between 0–40% and 70–80%, per capita fiscal expenditures will positively affect urban per capita income; however, the p value is low.

**Table 5:** The linear relationship between urban per capita income (dependent variable) and the explanatory variables under different poverty incidence.

Incidence of poverty	Serial number	Explanatory variables		Adjusted R squared	Serial number	Explanatory variables		Adjusted R squared
		Loans per capita	Fixed asset investment per capita			Public financial expenditures per capita	Fixed asset investment per capita	
**0–5%**	(39)	-0.069(0.316)	0.026(0.449)	-0.012	(58)	0.343(0.071)	-0.012(0.701)	0.02
**5–10%**	(40)	0.032(0.295)	0.086(0.000)	0.109	(59)	0.1(0.049)	0.068(0.007)	0.123
**10–15%**	(41)	0.045(0.181)	0.095(0.000)	0.071	(60)	-0.039(0.539)	0.119(0.000)	0.066
**15–20%**	(42)	0.067(0.026)	0.121(0.000)	0.132	(61)	0.106(0.095)	0.123(0.000)	0.127
**20–25%**	(43)	0.145(0.000)	0.141(0.000)	0.198	(62)	0.199(0.002)	0.145(0.000)	0.19
**25–30%**	(44)	0.063(0.118)	0.1(0.000)	0.135	(63)	0.326(0.000)	0.063(0.001)	0.22
**30–35%**	(45)	0.151(0.000)	0.008(0.69)	0.085	(64)	0.299(0.000)	0.019(0.253)	0.148
**35–40%**	(46)	0.208(0.000)	0.035(0.294)	0.191	(65)	0.279(0.009)	0.058(0.124)	0.128
**40–45%**	(47)	-0.025(0.562)	0.141(0.000)	0.12	(66)	-0.06(0.434)	0.143(0.000)	0.122
**45–50%**	(48)	-0.034(0.707)	0.166(0.009)	0.099	(67)	0.414(0.000)	0.059(0.178)	0.297
**50–55%**	(49)	0.456(0.000)	0.204(0.002)	0.558	(68)	-0.051(0.806)	0.372(0.000)	0.261
**55–60%**	(50)	-0.178(0.472)	0.241(0.067)	0.056	(69)	0.447(0.000)	0.083(0.236)	0.568
**60–65%**	(51)	0.893(0.434)	0.109(0.576)	0.516	(70)	-0.586(0.075)	0.486(0.007)	0.672
**65–70%**	(52)	0.274(0.004)	0.036(0.564)	0.748	(71)	0.343(0.174)	0.086(0.379)	0.563
**70–75%**	(53)	-0.017(0.946)	0.127(0.268)	0.741	(72)	0.149(0.114)	0.049(0.3)	0.814
**75–80%**	(54)	0.089(0.259)	0.09(0.025)	0.322	(73)	0.35(0.000)	0.02(0.534)	0.612
**80–85%**	(55)	0.478(0.026)	0.031(0.451)	0.496	(74)	-0.037(0.737)	0.095(0.255)	0.039
**85–90%**	(56)	0.596(0.055)	0.01(0.819)	0.503	(75)	0.187(0.028)	0.03(0.383)	0.559
**90–100%**	(57)	-0.692(0.668)	1.069(0.013)	0.878	(76)	0.279(0.047)	0.623(0.015)	0.957

According to Table 6, the estimation of Serial Nos. (81)—(87) shows that when the incidence of poverty is between 25–55%, per capita loans will have a positive impact on rural per capita income; however, the p value is low. The estimation of Serial Nos. (96)–(97) and (101)–(111) indicates that when the incidence of poverty is between 0–10% and 25–80%, per capita fiscal expenditures will positively influence rural per capita income; however, the p value is low.

**Table 6:** The linear relationship between Rural Per Capita Income (dependent variable) and the Explanatory variables under different poverty incidence.

Incidence of poverty	Serial number	Explanatory variables		Adjusted R squared	Serial number	Explanatory variables		Adjusted R squared
		Loans per capita	Fixed asset investment per capita			Public financial expenditure per capita	Fixed asset investment per capita	
**0–5%**	(77)	0.005(0.852)	0.026(0.064)	0.043	(96)	0.202(0.007)	0.015(0.219)	0.135
**5–10%**	(78)	0.026(0.083)	0.052(0.000)	0.18	(97)	0.085(0.000)	0.036(0.003)	0.224
**10–15%**	(79)	0.017(0.182)	0.039(0.000)	0.083	(98)	0.008(0.737)	0.042(0.000)	0.078
**15–20%**	(80)	0.002(0.887)	0.038(0.000)	0.06	(99)	0.036(0.144)	0.031(0.001)	0.065
**20–25%**	(81)	0.034(0.007)	0.049(0.000)	0.196	(100)	-0.016(0.432)	0.061(0.000)	0.179
**25–30%**	(82)	0.032(0.007)	0.02(0.000)	0.105	(101)	0.036(0.037)	0.021(0.000)	0.095
**30–35%**	(83)	0.039(0.001)	0.004(0.456)	0.079	(102)	0.044(0.006)	0.01(0.044)	0.064
**35–40%**	(84)	0.043(0.001)	0.006(0.503)	0.109	(103)	0.039(0.188)	0.016(0.132)	0.057
**40–45%**	(85)	0.046(0.002)	0.012(0.318)	0.147	(104)	0.047(0.075)	0.023(0.061)	0.094
**45–50%**	(86)	-0.03(0.175)	0.056(0.000)	0.152	(105)	0.065(0.004)	0.028(0.015)	0.216
**50–55%**	(87)	0.12(0.000)	0.03(0.182)	0.356	(106)	-0.097(0.137)	0.086(0.001)	0.14
**55–60%**	(88)	-0.012(0.868)	0.062(0.104)	0.061	(107)	0.045(0.148)	0.048(0.102)	0.122
**60–65%**	(89)	0.641(0.408)	-0.044(0.736)	0.002	(108)	-0.36(0.117)	0.202(0.066)	0.239
**65–70%**	(90)	0.093(0.02)	-0.019(0.486)	0.432	(109)	0.19(0.046)	-0.027(0.44)	0.355
**70–75%**	(91)	-0.054(0.616)	0.075(0.14)	0.745	(110)	0.058(0.171)	0.024(0.259)	0.795
**75–80%**	(92)	0.059(0.119)	0.000(0.992)	0.061	(111)	0.19(0.000)	-0.035(0.02)	0.554
**80–85%**	(93)	0.095(0.353)	0.006(0.785)	-0.049	(112)	-0.011(0.803)	0.021(0.535)	-0.167
**85–90%**	(94)	0.194(0.029)	0.003(0.827)	0.586	(113)	0.052(0.038)	0.011(0.27)	0.564
**90–100%**	(95)	1.158(0.012)	0.02(0.675)	0.913	(114)	-0.005(0.943)	0.184(0.166)	0.508

The results of the Piecewise Linear Regression Model show that the effects of financial and fiscal policies on economic development in counties had significant nonlinear characteristics. For example, when the incidence of poverty is relatively low or relatively high, finance has a very limited impact on per capita GDP, urban per capita income and rural per capita income. The influence increases when the incidence of poverty is at the medium level. Fiscal policy, in contrast, has more influence on per capita GDP, urban per capita income and rural per capita income when the incidence of poverty is relatively low or relatively high.

### Estimated results of the nonlinear model

To accurately capture how financial and fiscal policies influence the economy, this paper uses a nonlinear panel smooth transition model to estimate the relationships between different variables. The model is estimated by a Matlab software program provided by g. Colletaz (For more information, please see http://cchum-kvm-execandshare.in2p3.fr/CompanionSite/site.do?siteId=66). The author uploaded the original data to the website and then ran the data to obtain the model estimation results. Since the focus of this article is not on the PSTR estimation method, a detailed description of the specific estimation method is omitted.

#### Analysis of finance’s influence on the economy

**Fig 1:**
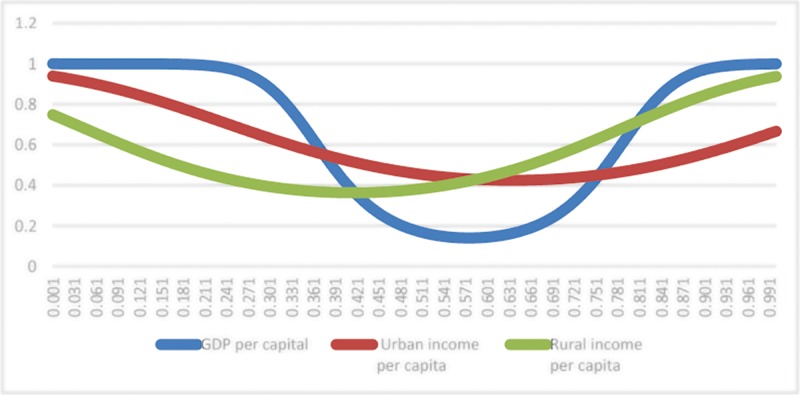
Transition functions under different explanatory variables in model (1).

The estimated results of Model (1) presented in Table 7 show that the transition of finance’s influence on per capita GDP occurs when the incidence of poverty is 6.3% and 57.1% at a relatively high speed, 18.3% and 44.9% at a relatively low speed for urban per capita income and 6.3% and 57.1% at relatively high speed for rural per capita income. Fig 1 shows the transformation function of financial influence under different explained variables.

**Table 7:** Estimation of nonlinear models (1) and (2).

		Linear part		Nonlinear part				
**Model(1)**	Explanatory variables	Loans per capita	Fixed asset investment per capita	Loans per capita	Fixed asset investment per capita	Positional parameter		Slope coefficient
	Parameter	$\beta_{1}^{b}$	$\beta_{2}^{b}$	$\beta_{3}^{b}$	$\beta_{4}^{b}$	C1	C2	*γ*
**Explained variable**	GDP per capita	0.778(0.287)***	0.337(0.203)*	-0.394(0.146)***	-0.171(0.062)***	0.063	0.571	56.782
	Urban income per capita	0.758(0.403)*	0.279(0.103)***	-0.49(0.195)***	-0.18(0.054)***	0.183	0.449	10.726
	Rural income per capita	0.275(0.155)*	0.086(0.051)*	-0.157(0.086)**	-0.049(0.136)	0.248	0.548	48.582
**Model(2)**	Explanatory variables	Public financial expenditures per capita	Fixed asset investment per capita	Public financial expenditures per capita	Fixed asset investment per capita	Positional parameter		Slope coefficient
	parameter	$\beta_{1}^{f}$	$\beta_{2}^{f}$	$\beta_{3}^{f}$	$\beta_{4}^{f}$	C1	C2	*γ*
**Explained variable**	GDP per capita	0.403(0.227)*	0.137(0.072)*	0.696(0.298)**	0.237(0.141)*	0.386	0.766	50.259
	Urban income per capita	-0.746(0.339)**	-0.17(0.105)*	2.732(0.279)***	0.622(0.368)*	0.435	0.837	7.539
	Rural income per capita	-0.058(0.029)**	-0.011(0.007)	0.65(0.117)***	0.127(0.538)	0.174	0.658	2.936

Note: 1. The numbers in brackets are the standard errors corresponding to the estimated parameters.

*, ** and *** indicate that the result is significantly different from zero under the significance level of 10%, 5% and 1%, respectively. 2. The model offers multiple choices to set up the number of positional parameters of the conversion function and the number of transition functions. Selected multiple choices and compared analysis results, we consider the results given by one conversion function and one transformation function that has two position parameters were the most actual. To save space, we did not list all the estimated results.

As Table 7 shows that the slope coefficient of the nonlinear part in the model is negative; the symmetry characteristics of transition functions indicate that when incidence of poverty is (*c*_1_+*c*_2_)/2, the influence of finance on the economy is the maximum, equaling $\beta_{1}^{b}+\beta_{3}^{b}*h_{z}(q=(c_{1}+c_{2})/2)$. The transition of finance’s influence on per capita GDP happens when c1 = 6.3% and c2 = 57.1%. Nevertheless, the symmetry of the transition functions indicate that when the incidence of poverty is c1 = 6.3% or c2 = 57.1%, finance has equal influence on per capita GDP, which is 0.581 ($\beta_{1}^{b}+\beta_{3}^{b}*h_{z}(q=c_{1}=6.3\%)=\beta_{1}^{b}+\beta_{3}^{b}*h_{z}(q=c_{2}=57.1\%)$). When the incidence of poverty is below c1 = 6.3% or above c2 = 57.1%, the influence of finance on per capita GDP is below 0.581; when the incidence of poverty is 6.3–57.1%, the influence is above 0.581. When the incidence of poverty is 31.7% ((*c*_1_+*c*_2_)/2), the influence reaches a maximum at 0.768 ($\beta_{1}^{b}+\beta_{3}^{b}*h_{z}(q=(c_{1}+c_{2})/2=31.7\%)$).

Regarding the influence of finance on urban per capita income, the transition happens when c1 = 18.3% and c2 = 44.9%. Given the symmetry of the transition functions, when the incidence of poverty is c1 = 18.3% or c2 = 44.9%, finance has equal influence on urban per capita income, which is 0.513 ($\beta_{1}^{b}+\beta_{3}^{b}*h_{z}(q=c_{1}=18.3\%)=\beta_{1}^{b}+\beta_{3}^{b}*h_{z}(q=c_{2}=44.9\%)$); when the incidence of poverty is below c1 = 18.3% or above c2 = 44.9%, the influence of finance on per capita GDP is below 0.513; when the incidence of poverty is 18.3–44.9%, the influence is above 0.513; and when the incidence of poverty is 31.6% ((*c*_1_+*c*_2_(/2), the influence reaches a maximum at 0.536 ($\beta_{1}^{b}+\beta_{3}^{b}*h_{z}(q=(c_{1}+c_{2})/2=31.6\%)$).

In terms of the influence of finance on rural per capita income, the transition takes place when c1 = 24.8% and c2 = 54.8%. Considering the symmetry of the transition functions, when the incidence of poverty is c1 = 24.8% or c2 = 54.8%, finance has equal influence on rural per capita income, which is 0.196 ($\beta_{1}^{b}+\beta_{3}^{b}*h_{z}(q=c_{1}=24.8\%)=\beta_{1}^{b}+\beta_{3}^{b}*h_{z}(q=c_{2}=54.8\%)$); when the incidence of poverty is below c1 = 24.8% or above c2 = 54.8%, the influence of finance on per capita GDP is below 0.196; when the incidence of poverty is 24.8–54.8%, the influence is above 0.196; when the incidence of poverty is 39.8% ((*c*_1_+*c*_2_)/2), the influence reaches a maximum at 0.235 ($\beta_{1}^{b}+\beta_{3}^{b}*h_{z}(q=(c_{1}+c_{2})/2=39.8\%)$).

#### Analysis of the influence of fiscal policies on the economy

**Fig 2:**
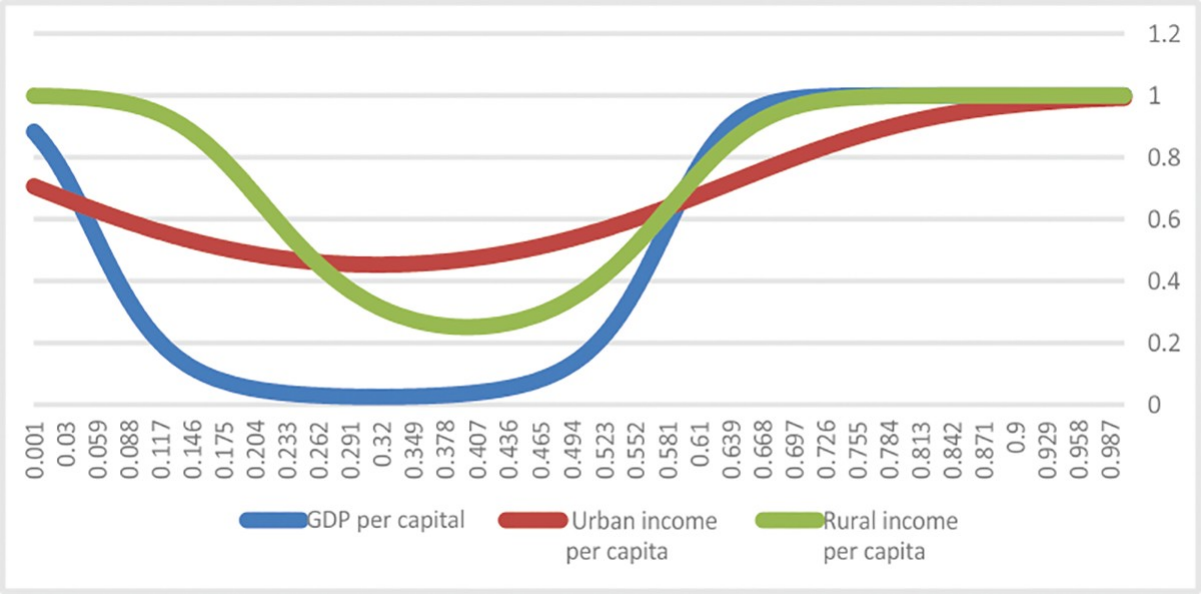
Transition functions under different explanatory variables in model (2).

The estimated results of Model (2) in Table 7 show that the transition of the fiscal policies’ influence on per capita GDP occurs when the incidence of poverty is 3.86% and 76.6% at a relatively high speed, 43.5% and 83.7.9% at a relatively low speed for urban per capita income and 17.4% and 65.8% at relatively high speed for rural per capita income. Fig 2 presents the influence of fiscal policies on the transition functions under different explanatory variables.

As Table 7 shows that the slope coefficient of the nonlinear part of the model is positive, the symmetry of the transition functions indicates that when the incidence of poverty is (*c*_1_+*c*_2_)/2, fiscal policies have their minimum influence on the economy, which is $\beta_{1}^{f}+\beta_{3}^{f}*h_{z}(q=(c_{1}+c_{2})/2)$. The transition of the influence of fiscal policies on per capita GDP happens when c1 = 38.6% and c2 = 76.6%. Nevertheless, the symmetry of the transition functions indicate that when the incidence of poverty is c1 = 38.6% or c2 = 76.6%, fiscal policies have equal influence on per capita GDP, which is 0.751 ($\beta_{1}^{f}+\beta_{3}^{f}*h_{z}(q=c_{1}=38.6\%)=\beta_{1}^{f}+\beta_{3}^{f}*h_{z}(q=c_{2}=76.6\%)$); when the incidence of poverty is below c1 = 38.6% or above c2 = 76.6%, the influence of fiscal policies on per capita GDP is above 0.751; when the incidence of poverty is 38.6–76.6%, the influence is below 0.751; when the incidence of poverty is 31.7% ((*c*_1_+*c*_2_)/2), the influence reaches its minimum at 0.5 ($\beta_{1}^{f}+\beta_{3}^{f}*h_{z}(q=(c_{1}+c_{2})/2=57.6\%)$).

Regarding the influence of fiscal policies on urban per capita income, the transition occurs when c1 = 43.5% and c2 = 83.7%. Given the symmetry of the transition functions, when the incidence of poverty is c1 = 43.5% or c2 = 83.7%, fiscal policies have equal influence on urban per capita income, which is 0.62 ($\beta_{1}^{f}+\beta_{3}^{f}*h_{z}(q=c_{1}=43.5\%)=\beta_{1}^{f}+\beta_{3}^{f}*h_{z}(q=c_{2}=83.7\%)$); when the incidence of poverty is below c1 = 43.5% or above c2 = 83.7%, the influence of fiscal policies on per capita GDP is above 0.62; when the incidence of poverty is 43.5–83.7%, the influence is below 0.62; when the incidence of poverty is 63.6% ((*c*_1_+*c*_2_)/2), the influence reaches the minimum at 0.413 ($\beta_{1}^{f}+\beta_{3}^{f}*h_{z}(q=(c_{1}+c_{2})/2=63.6\%)$).

In terms of the influence of fiscal policies on rural per capita income, the transition takes place when c1 = 17.4% and c2 = 65.8%. Considering the symmetry of the transition functions, when the incidence of poverty is c1 = 17.4% or c2 = 65.8%, fiscal policies have equal influence on rural per capita income, which is 0.267 (($\beta_{1}^{f}+\beta_{3}^{f}*h_{z}(q=c_{1}=17.4\%)=\beta_{1}^{f}+\beta_{3}^{f}*h_{z}(q=c_{2}=65.8\%)$); when the incidence of poverty is below c1 = 17.4% or above c2 = 65.8%, the influence of finance on per capita GDP is above 0.267; when the incidence of poverty is 17.4–65.8%, the influence is above 0.267; when the incidence of poverty is 41.6% ((*c*_1_+*c*_2_)/2), the influence reaches a minimum at 0.178 ($\beta_{1}^{b}+\beta_{3}^{b}*h_{z}(q=(c_{1}+c_{2})/2=39.8\%)$).

#### Comparison of the effects of financial and fiscal policies on the economy

We compare the effects of financial and fiscal policies on per capita GDP and found that, when the incidence of poverty is low or high, the marginal impact of changes in fiscal expenditures per capita 1 unit can change the GDP per capita more than 0.751, but the marginal effect of financial policies on per capita GDP is lower than 0.581; therefore, the marginal effect of fiscal expenditures on the per capita GDP is higher than the marginal effects of the financial policies. When the incidence of poverty is at the middle level, the impact of the marginal effect of financial policies on the GDP per capita is more than 0.581, and the highest can reach 0.768. The impact of the marginal effect of fiscal policies on the GDP per capita is lower than 0.751, and the lowest is only 0.5. Therefore, when the poverty rate is in the middle level, the marginal effect of financial policies on the per capita GDP is higher than that of the marginal effect of fiscal expenditures.

When we compare the effects of financial and fiscal expenditures on rural per capita income, a similar situation also appears. When the incidence of poverty is low or high, changing fiscal expenditures 1 unit makes the marginal effect of rural per capita income increases more than 0.267 units, while the marginal effect of financial policies lower than 0.196 units. Therefore, when the incidence of poverty is low or high, the marginal effect of fiscal expenditures on rural per capita income is more than the marginal effects of financial policies. When the incidence of poverty is in the middle level, the marginal effect of financial policies on city to rural per capita income will be more than 0.196 units and up to 0.235 units, but the marginal effects of fiscal policies are below 0.267; the lowest is only 0.178. As a result, when the poverty rate is in the middle level, the marginal effect of financial policies on rural per capita income is higher than the marginal effect of fiscal expenditures.

Summing up the above analysis, if the poverty rate is low or high and the marginal effect of financial policy on per capita GDP, urban per capita income, and rural per capita income will be lower than the marginal effect of fiscal expenditures. When the incidence of poverty is in the middle level, the marginal effects of financial policy will be more than the marginal effect of fiscal expenditures. When the incidence of poverty is high, the basic conditions in poverty areas are poor, and the ecological environment in which finance plays its role is not up to standard, so it is difficult for finance to play its role effectively. When the incidence poverty is at the intermediate level, the basic conditions in poor areas are relatively improved, and the ecological environment for finance can play a better role through marketization. Therefore, finance can play an effective role. After efforts have been made for poverty reduction, the incidence of poverty dropped to a lower level; it is hard to help people out of poverty through market-oriented means, namely, the market failure stage; at this moment, the government needs fiscal spending, etc. “the visible hand” to play a role, so in this phase, the marginal effect of fiscal expenditures is greater than that of finance.

#### Robustness test

(1) Nonlinear model transformation position

To further verify the rationality of the conversion position of the nonlinear model, we use the subsection regression method to estimate the model. However, the segmentation points were no longer segmented according to an equidistant poverty incidence rate but segmented according to the position points already found in the nonlinear model. The regression results are listed in Table 8. When the poverty incidence rate is low or high, the marginal effect of finance on the economic development level of poor counties is lower than that of fiscal expenditures, and the fitting effect of finance is slightly lower than that of fiscal expenditures. When the incidence of poverty is at a middle level, the marginal effect of finance on the economic development level of poor counties is slightly better than that of fiscal expenditures, and the model fitting is better. These results also confirm the results of the above nonlinear model.

**Table 8:** Piecewise linear regression results.

		GDP per capital		Urban per capita income		Rural per capita income	
Incidence of poverty		(1)	(2)	(3)	(4)	(5)	(6)
**Lower**	Per capita loans	0.097* (0.096)		0.021*(0.018)		0.026**(0.005)	
	Financial expenditures per capita		0.506**(0.305)		0.142***(0.021)		0.168***(0.012)
	Per capita fixed asset investment	0.146***(0.055)	0.057*(0.065)	0.055***(0.011)	0.065***(0.008)	0.051***(0.003)	0.038***(0.004)
	Adjusted R2	0.111	0.203	0.095	0.296	0.207	0.253
**Medium**	Per capita loans	0.261***(0.021)		0.257***(0.018)		0.205**(0.007)	
	Financial expenditures per capita		0.155**(0.033)		0.127***(0.047)		0.144**(0.009)
	Per capita fixed asset investment	0.291***(0.013)	0.321**(0.013)	0.038***(0.01)	0.018*(0.024)	0.017**(0.004)	0.011***(0.003)
	Adjusted R2	0.341	0.259	0.33	0.205	0.309	0.208
**Higher**	Per capita loans	0.256***(0.119)		0.120*(0.043)		0.113*(0.022)	
	Financial expenditures per capita		0.302(0.118)		0.206***(0.075)		0.322**(0.021)
	Per capita fixed asset investment	0.075*(0.045)	0.043*(0.062)	0.047**(0.019)	0.010*(0.046)	0.011*(0.008)	0.015*(0.016)
	Adjusted R2	0.103	0.301	0.108	0.309	0.103	0.302

Note: The positional parameters of the transformation function in the previous nonlinear regression model are cut-off points (i.e., the positional parameters c1 and c2 of the transformation function in Table 7), and the poverty incidence is divided into three grades: low, medium and high. For example, in the regression (1) in Table 8, per capita GDP is the dependent variable, the explanatory variables are per capita loans and per capita fixed asset investment, and the poverty incidence rate 6.3% and 57.1% are cut-off points (the two positional parameters shown in column 1 of model (1) in Table 7),the low, medium and high incidence of poverty respectively refers to the incidence of poverty is no more than 6.3%, the incidence of poverty is between 6.3% and 57.1%, and the incidence of poverty is more than 57.1%. 2. The values in brackets in Table 8 are the standard errors for estimating coefficients.3. In Table 8, *, ** and *** respectively indicate significant at the levels of 10%, 5% and 1%.

(2) Endogenous

This section discusses and corrects for the effect of endogeneity on the model estimation. The reason endogeneity is a problem is that there may be a two-way causal relationship between per capita GDP, urban per capita income, rural per capita income, per capita loan, per capita fiscal expenditures and per capita fixed asset investment, which are the explained variables in poor counties. Table 9 shows the results for the Granger causality test of the explanatory variables and explained variables. Loans per capital does not Granger cause GDP per capita, but the other explanatory variables are a Granger cause of the economy and income of the counties. However, conversely, poor areas of the economy and per capita income level are not a Granger cause of the explanatory variables. Therefore, the endogenous problem of this paper is not prominent.

**Table 9:** Granger causality test results between variables.

	The former does not Granger cause the latter	The latter does not Granger cause the former
**Per capita loans and per capita GDP**	0.373(0.688)	4.082(0.717)
**Per capita fiscal expenditures and per capita GDP**	20.070(0.002)	1.177(0.308)
**Per capita fixed asset investment and per capita GDP**	5.474(0.004)	19.986(0.361)
**Per capita loans and per capita urban income**	5.702(0.003)	0.429(0.650)
**Per capita fiscal expenditures and per capita urban income**	28.557(0.000)	0.593(0.552)
**Per capita fixed asset investment and per capita urban income**	4.786(0.008)	7.575(0.170)
**Per capita loans and rural per capita income**	4.146(0.016)	15.610(0.600)
**Per capita fiscal expenditures and rural per capita income**	15.987(0.000)	4.476(0.211)
**Per capita fixed asset investment and rural per capita income**	4.507(0.011)	6.232(0.242)

Note: The values in the table are F values of the Granger test, and the brackets are p values.

#### Summary of the results of the nonlinear models

Although both financial and fiscal policies have a positive influence on economic development, their influence varies for different levels of poverty. When the incidence of poverty is low or high, the impact of fiscal policies on economic development is stronger than that of finance; the converse is true when the poverty rate is at the medium level. For per capita GDP, for the transition of the effect of finance to occur, the incidence of poverty is 6.3% and 57.1%, respectively, and 38.6% and 76.6%, respectively, for fiscal policies. For per capita income in urban areas, the thresholds for the effect of finance are 18.3% and 44.9%, respectively, and 43.5% and 83.7%, respectively, for fiscal policies. For rural per capita income, the threshold points are 24.8% and 54.8%, respectively, for financial policies and 17.4% and 65.8%, respectively, for fiscal policies.

At different stages of poverty, the effects of financial and fiscal policies on economic development and poverty reduction are different. When the incidence of poverty is 0–6.3%, 6.3–57.1% and 57.1–100%, the effect of finance on per capita GDP is (0.428,0.518), (0.518, 0.768), and (0.384, 0.768), respectively. When the incidence of poverty is 0–38.6%, 38.6–76.6% and 76.6–100%, the effect of fiscal policies on per capita GDP is (0.751, 1.098), (0.5,0.751) and (0.751, 1.098), respectively.

The effect of finance on per capita income in cities is (0.411,0.513), (0.513, 0.536), and (0.271, 0.536) under an incidence of poverty of 0–6.3%, 6.3–57.1% and 57.1–100%, respectively. Under an incidence of poverty of 0–38.6%, 38.6–76.6% and 76.6–100%, the effect of fiscal policies on urban per capita income is (0.62, 1.821) (0.413, 0.62) and (0.413,1.076), respectively. When the incidence of poverty is 0–6.3%, 6.3–57.1%, and 57.1–100%, the effect of finance on rural per capita income is (0.118,0.196), (0.196, 0.235), (0.118, 0.235), respectively. When the poverty level is 0–38.6%, 38.6–76.6%, and 76.6–100%, the effect of fiscal policies on rural per capita income is (0.276,0.429) and (0.178, 0.276) and (0.178, 0.551), respectively.

## Conclusion

First, both financial and fiscal policies have a positive effect on promoting the economic development of poverty-stricken counties and thus reducing poverty. However, the promotion effect of financial and fiscal policies on the economic development of poverty-stricken counties will change with changes in the poverty level.

Second, the effect of financial and fiscal policies on the economic development of poor counties has obvious threshold characteristics. Using the incidence of poverty in counties to identify the threshold, financial and fiscal policies both have obvious changes at two special points of the poverty level, but the level of the threshold is different.

Third, although the level of the threshold is different, we can still use the two thresholds to divide the poverty level into three parts, namely, a low level of poverty, a medium level of poverty, and a high level of poverty. We find that at the three different stages of poverty, financial and fiscal policies have a certain complementary influence; at the low or high levels of poverty, the fiscal policies had a significantly larger effect than the financial policies. However, when the poverty level is in the middle, the effect of finance policy on the economic and income level of poor counties is significantly greater than that of fiscal policy.

Based on the above research conclusions, we suggest that different combinations of policies should be implemented according to the changes in the poverty situation. For the phase of high poverty level in poor areas, while implementing financial policy on poverty reduction, we should give priority to the role of fiscal policy on poverty reduction. When the poverty level in poor areas is in the middle, while continuing to implement fiscal poverty reduction policies, we should focus on increasing financial support on poverty reduction. When the poverty level is pushed down to a lower level, that is to say, the finishing stage of poverty alleviation, while maintaining the financial poverty alleviation policy, we should turn to increasing fiscal support on poverty reduction.

Income inequality and external shocks will affect the effect of financial policies and fiscal policies on poverty reduction. Due to the limitations of space and data availability, this paper did not consider this, which is our future research direction.

## Supporting information

S1 Dataset (XLS)
